# Influence of a biliary stent in patients with advanced pancreatic cancer treated with modified FOLFIRINOX

**DOI:** 10.1097/MD.0000000000032150

**Published:** 2022-12-09

**Authors:** Akie Yoshikawa-Kimura, Koichi Taira, Hirotsugu Maruyama, Yuki Ishikawa-Kakiya, Masafumi Yamamura, Kojiro Tanoue, Atsushi Hagihara, Sawako Uchida-Kobayashi, Masaru Enomoto, Kenjiro Kimura, Shogo Tanaka, Ryosuke Amano, Shigekazu Takemura, Satoko Ohfuji, Fumio Tanaka, Yasuaki Nagami, Yasuhiro Fujiwara

**Affiliations:** a Department of Gastroenterology, Osaka City University Graduate School of Medicine, Abeno-ku, Osaka, Japan; b Department of Gastroenterology, Osaka Metropolitan University Graduate School of Medicine, Abeno-ku, Osaka, Japan; c Department of Hepatology, Osaka Metropolitan University Graduate School of Medicine, Abeno-ku, Osaka, Japan; d Department of Hepato-Biliary-Pancreatic Surgery, Osaka Metropolitan University Graduate School of Medicine, Abeno-ku, Osaka, Japan; e Department of Public Health, Osaka Metropolitan University Graduate School of Medicine, Abeno-ku, Osaka, Japan.

**Keywords:** biliary stent, malignant biliary obstruction, modified FOLFIRINOX, pancreatic cancer

## Abstract

Endoscopic biliary drainage is the recommended 1^st^-line treatment for malignant biliary obstruction. Although a high incidence of febrile neutropenia has been reported in patients treated with FOLFIRINOX and a biliary stent, it remains unknown whether the biliary stent contributes to patient survival. Thus, we aimed to elucidate the effects of biliary stents on the survival of patients with advanced pancreatic cancer treated with modified FOLFIRINOX (mFFX). We retrospectively reviewed medical charts of patients with advanced pancreatic cancer treated with mFFX between January 2014 and April 2020. We compared the overall survival (OS) of patients with and without biliary stent during mFFX treatment and examined the independent effect on mortality using propensity score matching. Overall, we included 89 patients (stent group, n = 24; non-stent group, n = 65). The proportion of patients with pancreatic head cancer was significantly higher in the stent group than in the non-stent group (*P *< .01). Stratification analysis in patients with pancreatic head cancer revealed that OS was significantly shorter in the stent group than in the non-stent group (*P *= .03). After propensity score matching, 19 pairs of patients in each group were analyzed. The stent group revealed a significantly shorter survival than the non-stent group (median OS, 10.3 vs 24.9 months; *P *< .01). The incidences of febrile neutropenia (*P *= .01) and biliary tract-related events that required biliary stenting or stent replacement (*P *< .01) were significantly higher in the stent group than in the non-stent group. Stent insertion was an independent risk factor for overall mortality. Biliary stents may reduce survival in patients with advanced pancreatic cancer. The rate of febrile neutropenia was higher in the stent group than in the non-stent group. There is a need to assess the patient’s condition with discretion and develop a treatment strategy with short prognosis in mind after stent insertion.

## 1. Introduction

Pancreatic cancer is the 4^th^-leading cause of cancer-related deaths in the United States and is associated with high morbidity and mortality in Japan.^[1]^ Malignant biliary obstruction (MBO) occurs in approximately 64% to 77% of cases of pancreatic cancer.^[2]^ Endoscopic biliary drainage (EBD) is the 1^st^ choice to improve MBO.^[3]^ Adequate biliary drainage can prevent hepatobiliary dysfunction and liver failure, as well as improve a patient’s quality of life. It is crucial to manage biliary tract-related events, such as obstructive jaundice and cholangitis, caused by MBO in patients with advanced pancreatic cancer who receive chemotherapy. Although there is no large data on the correlation between chemotherapy and EBD, a previous study demonstrated that febrile neutropenia (FN) was highly prevalent in patients who were treated with FOLFIRINOX (FOLFIRINOX [a combination of 5-fluorouracil, oxaliplatin, irinotecan, and leucovorin] [FFX]; a combination of 5-fluorouracil [5FU], oxaliplatin, irinotecan, and leucovorin) -related regimen and a biliary stent, compared with those without biliary stent^[4,5]^; moreover, it was reported that the initial biliary stent insertion was an independent risk factor for FN in patients treated with FFX.^[6]^ Therefore, it is important to administer a combination chemotherapy, such as FFX-related regimens, while paying attention to serious side effects, such as severe myelosuppression and FN. However, it remains unknown whether a biliary stent before and during chemotherapy affects survival.

Gemcitabine (GEM) monotherapy has traditionally been the 1^st^-line chemotherapy for advanced pancreatic cancer^[7]^; however, combination therapy with FFX and GEM plus nab-paclitaxel is superior to GEM monotherapy and is currently the standard 1^st^-line chemotherapy for advanced pancreatic cancer.^[8,9]^ The toxicity of FFX is severe; therefore, modified FOLFIRINOX (modified FOLFIRINOX [mFFX]; reduced doses of 5FU bolus and irinotecan) is used frequently in Japan.^[10]^ The national comprehensive cancer network guidelines recommend FFX or mFFX and GEM plus nab-paclitaxel as the 1^st^-line systemic chemotherapy and subsequent therapy for relatively healthy patients with locally advanced, metastatic, and recurrent pancreatic cancer.^[11]^ Therefore, mFFX is a highly effective and key regimen in pancreatic cancer.

This study aimed to elucidate the effect of biliary stents on the survival of patients with advanced pancreatic cancer treated with mFFX.

## 2. Methods

### 2.1. Patients

Between January 2014 and April 2020, we enrolled 106 patients with advanced pancreatic cancer who received mFFX at our institution and retrospectively reviewed their medical records and databases.

The following were the inclusion criteria: pathological and clinical diagnosis of locally advanced, metastatic, or recurrent pancreatic adenocarcinoma; mFFX administration at least once as the 1^st^-line or second-line treatment; eastern cooperative oncology group performance status (ECOG PS) of 0 to 2; and consent to receive chemotherapy.

The following were the exclusion criteria: a relapse within 6 months after the surgery; the development of severe complications and other organ dysfunctions; and missing data.

Among 106 patients enrolled in the study, 17 relapsed within 6 months of surgery; their recurrence occurred during S-1 adjuvant chemotherapy and these patients were excluded. No patients were excluded due to severe complications, organ dysfunction, or missing data. Among the remaining 89 patients who fulfilled the inclusion criteria, 24 (27.0%) were treated with EBD (stent group) and 65 (73.0%) were not treated with EBD (non-stent group).

In terms of the timing of biliary stent insertion, patients who underwent stent insertion both before and during mFFX treatment were included, and patients who underwent stent insertion after mFFX treatment were excluded.

### 2.2. Treatment

Patients were treated with mFFX every 2 weeks as follows: a 2-hour intravenous infusion of oxaliplatin (85 mg/m^2^), infusion of l-leucovorin (200 mg/m^2^), 90-minute intravenous infusion of irinotecan (150 mg/m^2^), and continuous 46-hour intravenous infusion of 5FU (2400 mg/m^2^).^[10]^ All patients routinely received palonosetron, aprepitant, and dexamethasone for emesis prophylaxis. Granulocyte colony-stimulating factor was not included in the primary prophylaxis. We defined second-line use of mFFX as the use of mFFX following GEM-based chemotherapy as the 1^st^-line treatment. We allowed dose reduction of mFFX in the 1^st^ cycle because of myelosuppression due to 1^st^-line chemotherapy. The treatment was continued until disease progression, unacceptable toxicity, or patient refusal. The respective physician determined the dose of any drug according to the patient’s general condition and adverse events.

EBD was performed when MBO was diagnosed, when obstructive jaundice or cholangitis was predicted secondary to MBO, or when stent dysfunction, such as stent obstruction, stent migration, and cholangitis, occurred even after stent insertion. Obstructive jaundice was diagnosed based on laboratory investigations and biliary dilation on computed tomography images. Cholangitis was defined as the elevation of serum liver and/or biliary enzyme levels and the presence of typical symptoms, including fever. Initial biliary stent insertion was defined as stent placement in the bile duct for MBO, which was observed on imaging or blood tests before the initiation of mFFX treatment. An expert endoscopist selected the device of choice for EBD in gastroenterology in each case.

All patients underwent computed tomography for tumor staging regularly to determine the effects of chemotherapy. Diagnostic staging and resectability classifications were based on the 7^th^ edition of the Japanese Classification of Pancreatic Cancer.^[12]^

### 2.3. Data collection and evaluation

Tumor responses were evaluated according to the Response Evaluation Criteria in Solid Tumors, version 1.1. Toxicity was assessed using the Common Terminology Criteria for Adverse Events version 4.1. The date of death and that of the last follow-up were reviewed to estimate the survival time. Survival and follow-up data were reviewed by the study investigators until September 1, 2020. Overall survival (OS) was defined as the interval from the start of chemotherapy for pancreatic cancer until death.

The study protocol was approved by the ethics committee of the Osaka City University Graduate School of Medicine (No. 2020-149) on August 26, 2020. This study was conducted according to the Declaration of Helsinki and all patients provided written informed consent before participating in the study.

### 2.4. Endpoints

We defined OS as the main outcome to evaluate the effects of biliary stents on chemotherapy, with the mFFX regimen. We also evaluated the adverse events and risk factors for mortality of the mFFX regimen according to the presence of a biliary stent.

### 2.5. Statistical analysis

All statistical analyses were performed using EZR (Saitama Medical Center, Jichi Medical University, Saitama, Japan), which is a graphical user interface for R (The R Foundation for Statistical Computing, Vienna, Austria); specifically, it is a modified version of R commander designed to add statistical functions frequently used in biostatistics.^[13]^ Categorical variables were analyzed using the chi-squared or Fisher’s exact test, and continuous variables were compared using the unpaired *t* test or Mann Whitney U test. OS was estimated using the Kaplan-Meier method, and the Kaplan-Meier curves were compared using the log-rank test.

Propensity score matching was used to minimize selection bias and balance the variables. Propensity scores were estimated using a logistic regression model with location of pancreatic cancer and body mass index (BMI) as covariates. A 1-to-1 nearest-neighbor matching algorithm with an optimal of 0.2 without replacement was used to generate 19 pairs of patients. Propensity score matching was performed using the MatchIt package in R version 1.54. The variables that could affect mortality were estimated by calculating the hazard ratio (HR) and 95% confidence interval (CI) by Cox proportional hazard model. Long-term outcomes were assessed using the log-rank test and the Kaplan-Meier method. All statistical tests were 2-sided, and a *P* value < 0.05 was considered statistically significant.

## 3. Results

### 3.1. Patient characteristics

A flowchart indicating the advanced pancreatic cancer patients who received modified FOLFIRINOX in our institution has been summarized in Figure S1, Supplemental Digital Content, http://links.lww.com/MD/I54. The patient characteristics are summarized in Table 1.

**Table 1:** Patient characteristics according to stenting.

	stent (n = 24)	non-stent (n = 65)	*p* value
Age, median [range], yr	65 [43–74]	66 [28–78]	.45
Sex, male, n (%)	14 (58.3)	39 (60.0)	1
ECOG PS, n (%)			.2
0	14 (58.3)	33 (50.8)	
1	9 (37.5)	32 (49.2)	
2	1 (4.2)	0	
Location of pancreatic tumor, n (%)			<.01
Head	20 (83.3)	20 (30.8)	
Body/ tail	4 (16.7)	45 (69.2)	
Distant metastasis			
Yes, n (%)	21 (87.5)	56 (86.2)	1
Metastatic site, n (%)			
Liver	14 (58.3)	25 (38.5)	
Lung	3 (12.5)	16 (24.6)	
Peritonium	3 (12.5)	23 (35.4)	
Lymph node	11 (45.8)	23 (35.4)	
Other (locally recurrence etc)	3 (12.5)	20 (30.8)	
Resectable classification, n (%)			.005
UR-M	20 (83.3)	29 (44.6)	
UR-LA	1 (4.2)	9 (13.8)	
recurrence	3 (12.5)	27 (41.5)	
UGT1A1, n (%)			.48
Wild type	8 (33.3)	31 (47.7)	
6 hetero/ 28 hetero	8 (33.3)	17 (26.2)	
6 homo	0	2 (3.1)	
BMI, kg/m^2^ [range]	19.5 [14.8–26.2]	21.2 [15.8–31.4]	.03
CEA, ng/mL [range]	9.10 [1.5–304.3]	6.55 [0.8–262.2]	.36
CA19-9, IU/mL [range]	366.5 [7–479965]	461.5 [2–1141615]	.81
mFFX as 1st/ 2nd line treatment	11 / 13	26/ 39	
First line treatment, n (%)			
GnP	10 (41.7)	35 (53.8)	
GS	2 (8.3)	3 (4.6)	
GEM monotherapy	1 (4.2)	1 (1.5)	
mFFX	11 (45.8)	26 (40.0)	.64

BMI = body mass index, CA19-9 = carbohydrate antigen 19-9, CEA = carcinoembryonic antigen, ECOG PS = Eastern cooperative Oncology Group performance status, GEM = Gemcitabine, GnP = Gemcitabine plus nab-paclitaxel, GS = Gemcitabine plus S-1 (tegafur/ gimeracil/ oteracil), mFFX = modified FOLFIRINOX, UGT1A1 = uridine diphosphate glucuronosyltransferase 1A1.

The overall median age was 66 years, 53 (59.6%) patients were male, and 88 (98.9%) had an ECOG PS of 0 to 1. The primary tumor site was the head of the pancreas in 40 (45.0%) patients, and 19 (21.3%) received an initial biliary stent. Uridine diphosphate glucuronosyltransferase (UGT) genetic polymorphisms were classified as wild-type (43.8%), heterozygous (28.1%), and homozygous (2.2%), while data were unavailable for some patients (25.8%).

The proportion of patients with pancreatic head (Ph) cancer was significantly higher in the stent group than in the non-stent group (83.3% vs 30.8%; *P* < .01). The pretreatment BMI was significantly lower in the stent group than in the non-stent group (median, 19.5 vs 21.2 kg/m^2^; *P* = .03). Other parameters such as age, sex distribution, ECOG PS, percentage of distant metastasis, pretreatment serum levels of carcinoembryonic antigen and carbohydrate antigen (CA) 19-9, and the proportion of mFFX as the 1^st^-line treatment were not significantly different between the 2 groups.

The stent group included 19 patients who underwent stent insertion before mFFX treatment (plastic stent, n = 7; self-expandable metallic stent [SEMS], n = 12) and 5 who underwent stent insertion during mFFX treatment (plastic stent, n = 1; SEMS, n = 4). Among the 12 patients who underwent SEMS insertion before mFFX, 2 required replacement during mFFX treatment, while 10 could continue mFFX without needing a replacement. Four patients who underwent SEMS insertion during mFFX treatment could also continue the treatment without needing a replacement.

### 3.2. Toxicity

Table 2 summarizes the severe adverse events (grade ≥ 3) related to mFFX administration.

**Table 2:** Grade ≥3 severe adverse events according to mFFX.

Event, n (%)	Stent (n = 24)	Non-stent (n = 65)	*p* value
Neutropenia	9 (37.5)	21 (32.3)	.80
Anemia	1 (4.2)	1 (1.5)	.47
Thrombocytopenia	0	2 (3.1)	1.00
Febrile neutropenia	6 (25.0)	3 (4.6)	.01
Fatigue	2 (8.3)	2 (3.1)	.29
Decreased appetite	1 (4.2)	2 (3.1)	1.00
Diarrhea	2 (8.3)	0	.07
Constipation	0	0	.00
Nausea	0	0	.00
Peripheral sensory neuropathy	0	2 (3.1)	1.00
Gastrointestinal perforation	1 (4.2)	0	.27
Obstructive jaundice or cholangitis * during mFFX administration	9 (37.5)	0	<.01
Obstructive jaundice or cholangitis * during overall survival	14 (58.3)	1 (1.5)	<.01

mFFX, modified FOLFIRINOX.

* Obstructive jaundice or cholangitis that is not in febrile neutropenia but requires biliary stenting or stent replacement.

The rate of neutropenia was not significantly different between the 2 groups (stent group vs non-stent group; 37.5% vs 32.3%, *P* = .80), but the rate of FN was significantly higher in the stent group than in the non-stent group (25.0% vs 4.6%; *P* = .01). We focused on the frequency of biliary tract-related events, such as obstructive jaundice or cholangitis caused by MBO that was not FN but required treatment with EBD. Recurrent biliary obstruction after EBD was counted as biliary-related events. The proportion of events requiring treatment with 2 or more EBDs was significantly higher in the stent group than in the non-stent group, both during mFFX (37.5% vs 0%, *P* < .01) and during OS (58.3% vs 1.5%, *P* < .01). One patient in the non-stent group died of septic shock due to cholangitis without biliary drainage because he was in too poor of a condition to be treated aggressively. In this study, none of the patients died from severe complications or procedures related to EBD. No patient was treated with endoscopic ultrasound-guided biliary drainage.

### 3.3. Efficacy of mffx

The best tumor response to mFFX is summarized in Table 3.

**Table 3:** Best tumor response to mFFX administration.

	Total (n = 89)	Stent (n = 24)	Non-stent (n = 65)	*p* value
Complete response	0 (0%)	0 (0%)	0 (0%)	
Partial response	10 (11.2%)	3 (12.5%)	7 (10.8%)	
Stable disease	38 (42.7%)	7 (29.2%)	31 (47.7%)	
Progressive disease	23 (25.8%)	6 (25.0%)	17 (26.2%)	
Not evaluable	18 (20.2%)	8 (33.3%)	10 (15.4%)	
DCR (%)	53.9	41.7	58.5	.23

CR = complete response. DCR, disease control rate (CR+PR+SD), NE = not evaluable, PD = progressive disease, PR = partial response, SD = stable disease.

Overall, 10 patients achieved partial response and 38 showed stable disease; therefore, the total disease control rate was 54%. There was no significant difference in disease control rate between the 2 groups (stent group vs non-stent group; 41.7% vs 58.5%; *P* = .23).

**Figure 1.**
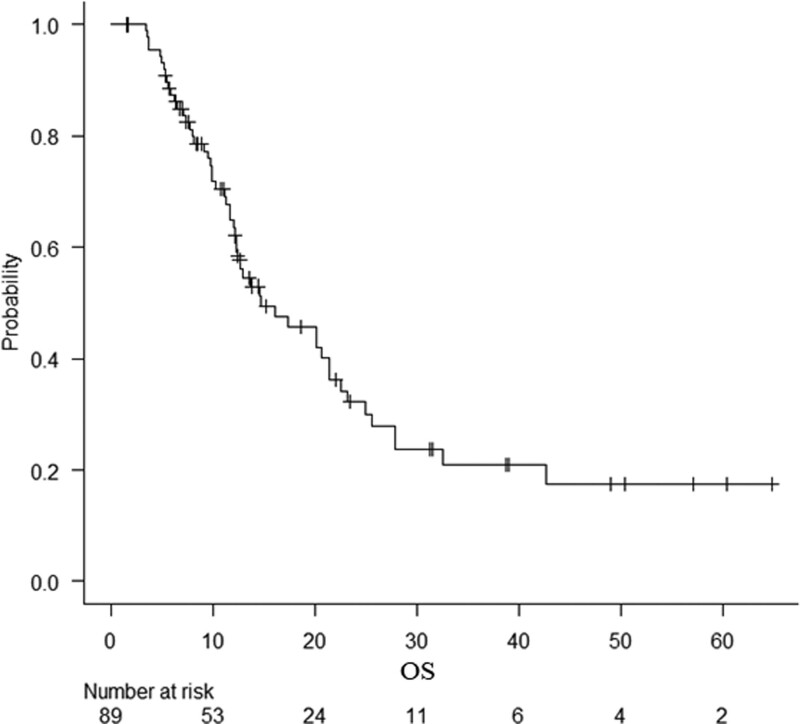
Kaplan-Meier estimates of overall survival (OS) in 89 patients with advanced pancreatic cancer who were treated with modified FOLFIRINOX. The median OS was 14.7 months (95% CI: 12.4–21.4). CI = confidence interval, OS = overall survival.

**Figure 2.**
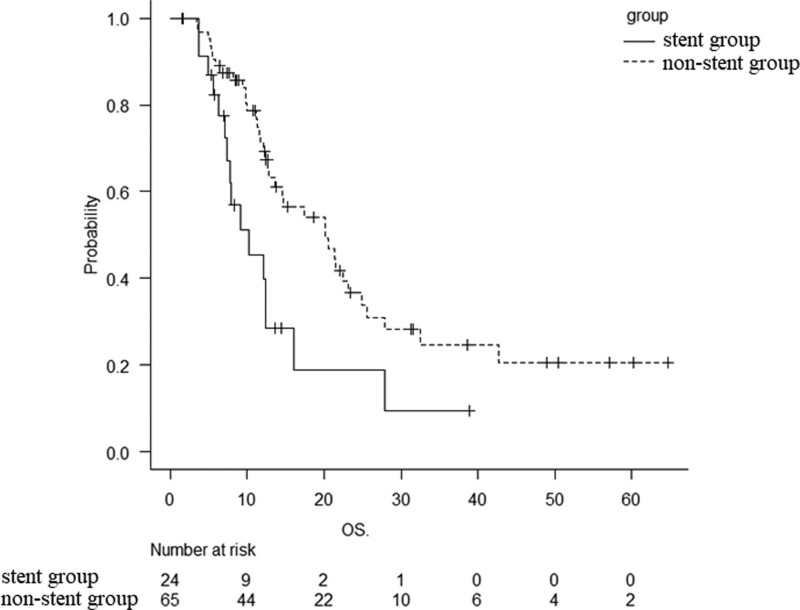
Overall survival (OS) in 89 patients (24 in the stent group and 65 in the non-stent group) with 1^st^- or second-line mFFX treatment. The median OS was 10.3 months (95% CI: 7.1–12.5) and 20.2 months (95% CI: 12.9–24.9) in the stent group and non-stent group, respectively (*P* < .01). CI = confidence interval, OS = overall survival.

The overall median OS was 14.7 months (95% CI: 12.4–21.4) (Fig. 1). The comparison of the median OS between the 2 groups, regardless of whether mFFX was 1^st^- or second-line treatment, revealed a shorter survival in the stent group than in the non-stent group (10.3 vs 20.2 months; *P* < .01) (Fig. 2). Since the location of pancreatic cancer greatly affects the requirement of a biliary stent, stratified analysis was performed only in patients with Ph cancer, revealing that the median OS was still shorter in the stent group than in the non-stent group (12.1 vs 24.9 months; *P* = .03). On the other hand, the median OS was also shorter in the stent group than in the non-stent group in patients with pancreatic body/tail (Pbt) cancer (9.7 vs 17.4 months; *P* = .02) (Figure S2, Supplemental Digital Content, http://links.lww.com/MD/I55).

**Figure 3.**
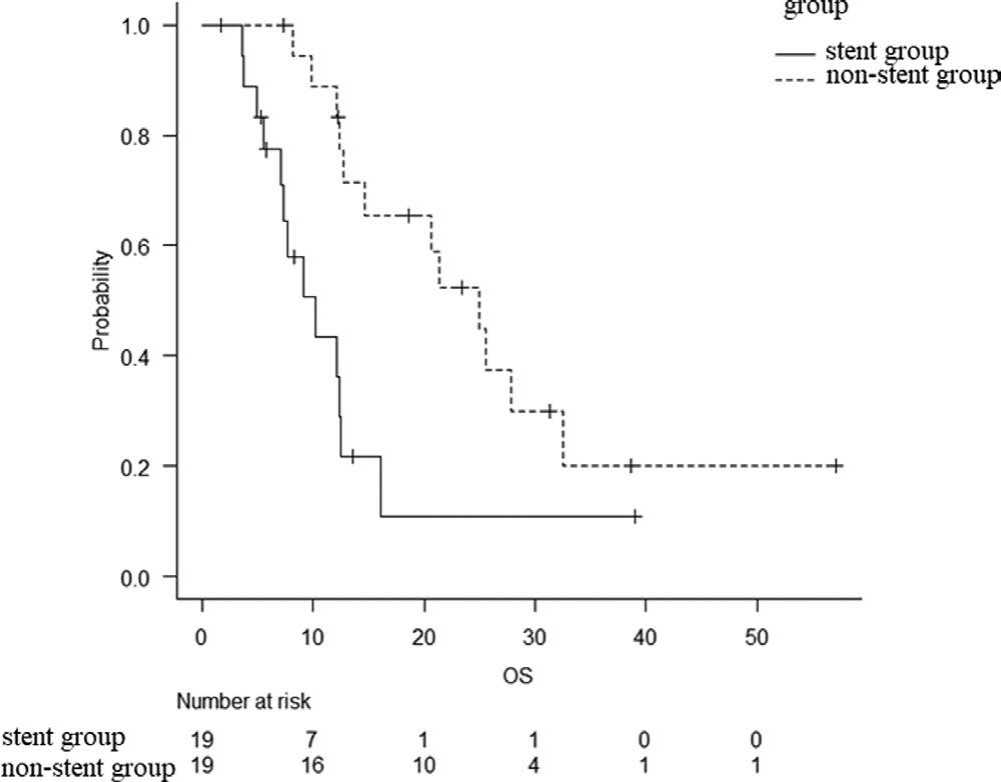
Overall survival (OS) after propensity score matching. The median OS was 10.3 months (95% CI: 7.1–12.5) and 24.9 months (95% CI: 12.7–32.6) in the stent group and non-stent group, respectively (*P* < .01). CI = confidence interval, OS = overall survival.

The location of the pancreatic tumor and BMI differed significantly in the baseline characteristics between the 2 groups before matching. After matching, none of the factors differed significantly in the baseline characteristics among the 19 matched pairs of patients (Table 4). After propensity score matching, the median OS was 10.3 months in the stent group and 24.9 months in the non-stent group (*P* < .01) (Fig. 3). The stent group demonstrated a significantly shorter survival time than the non-stent group before and after matching.

**Table 4:** Patient characteristics before and after propensity score matching.

	Before matching (n = 89)			After matching (n = 38)		
	stent (n = 24)	non-stent (n = 65)	p value	stent (n = 19)	non-stent (n = 19)	*p* value
Age (mean [SD]), y	62.1 (8.6)	63.5 (9.5)	0.51	61.0 (7.8)	65.1 (7.6)	.1
Sex, male, n (%)	14 (58.3)	39 (60.0)	1	10 (52.6)	10 (52.6)	1
ECOG PS, n (%)						
0	14 (58.3)	33 (50.8)	0.69	11 (57.9)	7 (36.8)	.33
1/ 2	10 (41.7)	32 (49.2)		8 (42.1)	12 (63.2)	
Location of pancreatic tumor, n (%)						
Head	20 (83.3)	20 (30.8)	< 0.001	15 (78.9)	15 (78.9)	1
Body/ tail	4 (16.7)	45 (69.2)		4 (21.1)	4 (21.1)	
Distant metastasis						
Yes, n (%)	21 (87.5)	56 (86.2)	1	17 (89.5)	16 (84.2)	1
BMI, kg/m^2^ (mean (SD))	20.0 (2.6)	21.4 (2.9)	0.04	20.7 (2.4)	20.7 (2.3)	1
CEA, ng/mL (mean (SD))	35.7 (72.0)	21.0 (44.0)	0.25	42.1 (80.0)	12.3 (11.3)	.14
CA19-9, IU/mL (mean (SD))	28915.8 (99643.8)	31567.8 (148417.5)	0.94	28857.4 (109518.9)	9050.3 (27505.5)	.45
Treatment line						
mFFX as first line, n (%)	11 (46.8)	26 (40.0)	0.8	10 (52.6)	5 (26.3)	.18

BMI = body mass index, ECOG PS = Eastern cooperative Oncology Group performance status,CA19-9 = carbohydrate antigen 19-9, CEA = carcinoembryonic antigen, mFFX = modified FOLFIRINOX.

### 3.4. Risk factors for mortality

In the Cox proportional hazard analysis (Table 5), stent insertion was significantly associated with shorter survival before matching (HR, 2.22; 95% CI: 1.22–4.04; *P* < .01).

**Table 5:** Risk factors for mortality by crude Cox proportional hazards model before and after propensity score-matching.

		Before matching (n = 89)				After matching (n = 38)			
		n	Case (%)	Crude HR (95% CI)	p value	n	Case (%)	Crude HR (95% CI)	*p* value
Stent	no	65	37 (56.9)	1.00	< 0.01	19	12 (63.2)	1.00	<.01
	yes	24	16 (66.7)	2.22 (1.22–4.04)		19	13 (68.4)	3.16 (1.39–7.19)	
Age		89	53 (59.6)	0.97 (0.94–1.00)	0.06	38	25 (65.8)	0.96 (0.91–1.01)	.14
Sex	male	53	30 (56.6)	1.00	0.24	20	14 (70.0)	1.00	.59
	female	36	23 (63.9)	1.39 (0.80–2.40)		18	11 (61.1)	0.81 (0.36–1.78)	
Location	head	40	22 (55.0)	1.00	0.78	30	18 (60.0)	1.00	.68
	body/ tail	49	31 (63.3)	1.08 (0.62–1.87)		8	7 (87.5)	1.20 (0.50–2.89)	
ECOG PS	0	47	23 (48.9)	1.00	0.44	18	9 (50.0)	1.00	.53
	1/ 2	42	30 (71.4)	1.24 (0.72–2.13)		20	16 (80.0)	1.30 (0.57–2.95)	
Distant metastasis	no	12	8 (66.7)	1.00	0.19	5	4 (80.0)	1.00	.01
	yes	77	45 (58.4)	0.60 (0.28–1.29)		33	21 (63.6)	0.18 (0.05–0.68)	
Treatment line of mFFX	1st	37	20 (54.1)	1.00	0.22	15	8 (53.3)	1.00	.62
	2nd	52	33 (63.5)	0.70 (0.40–1.23)		23	17 (73.9)	0.80 (0.34–1.88)	
BMI		89	53 (59.6)	0.95 (0.86–1.05)	0.27	38	25 (65.8)	0.94 (0.79–1.13)	.52
CEA		89	53 (59.6)	1.00 (1.00–1.01)	0.28	38	25 (65.8)	1.01 (0.99–1.01)	.09
CA19-9		89	53 (59.6)	1.00 (0.99–1.01)	0.87	38	25 (65.8)	1.00 (1.00–1.01)	.65

BMI = body mass index, ECOG PS = Eastern cooperative Oncology Group performance status; CA19-9 = carbohydrate antigen 19-9, CEA = carcinoembryonic antigen, mFFX = modified FOLFIRINOX.

After matching, stent insertion was significantly associated with shorter survival (HR, 3.16; 95% CI: 1.39–7.19; *P* < .01). Distant metastasis was significantly associated with longer survival (HR, 0.18; 95% CI: 0.05–0.68; *P* = .01).

## 4. Discussion

We demonstrated that patients with pancreatic cancer who received mFFX and required biliary stent insertion had significantly shorter survival than did those who did not need biliary stent insertion. We also showed that stent insertion was an independent risk factor for overall mortality, even after adjusting for other confounding factors using propensity score matching. Moreover, we found that the incidence of severe adverse events, such as FN, was higher in the stent group than in the non-stent group; however, the tumor response to mFFX was not significantly different between the 2 groups. To our knowledge, this is the 1^st^ report on the effects of biliary stents on survival in patients with pancreatic cancer and those undergoing mFFX treatment using propensity matching score analysis.

Ph cancer is more likely to cause biliary obstruction and necessitate a stent than Pbt cancer. However, stent insertion is required in Pbt cancer for large tumors or liver hilar lymphadenopathy; in this study, only 4 cases of Pbt cancer required stenting for these reasons. Several reports have demonstrated the association between tumor location and clinical outcomes with controversial results.^[14–16]^ Meng et al reported that Pbt cancer had a better prognosis than Ph cancer in cases of surgical resection.^[15]^ Conversely, a recent meta-analysis showed that Ph cancer is predictive of a better prognosis.^[16]^ However, previous reports found it difficult to compare the patient background data. To solve this problem, we compared OS since the initiation of treatment between the stent and non-stent groups irrespective of whether mFFX was administered as 1^st^- or second-line treatment, performed stratification analysis only in patients with Ph cancer, and used propensity score matching to reduce the selection bias. The results showed that the stent group had significantly shorter survival than the non-stent group. Furthermore, there was no significant difference in the median OS between Ph and Pbt cancer (16.1 vs 13.6 months; *P *= .78) (Figure S3, Supplemental Digital Content, http://links.lww.com/MD/I56). These results revealed no significant difference in survival among patients with different pancreatic cancer locations irrespective of stent insertion, thus suggesting that biliary stents have a large effect on the prognosis of these patients.

In our study, we focused on adverse events related to FN and severe neutropenia to investigate the association between biliary stents and mFFX treatment. The proportions of FN and biliary tract-related events that required biliary stenting or stent replacement were significantly higher in the stent group than in the non-stent group, whereas the rate of severe neutropenia was not significantly different between the groups. In the stent group, more than half of the patients required stent replacements at least once due to MBO; however, it was difficult to follow-up and assess whether biliary tract-related events directly resulted in death. Here, most deaths were caused by the primary disease. From the results in Table 2, it was considered that the stent group had more frequent onsets of FN, obstructive jaundice, and cholangitis, which caused a delay in chemotherapy and decrease in physical strength and immunity, resulting a shorter survival. In a phase II study of mFFX in Japan, the rate of Grade 3 to 4 neutropenia was 47.8%, and FN was noted in 8.7% of patients.^[10]^ In another prospective study on mFFX in Japan, the overall Grade 3 to 4 rate of neutropenia was 83.9% and that of FN was 16.1%; additionally, FN was noted in 50% of patients with biliary stent placement at baseline.^[4]^ From these results, it can be considered that biliary tract-related events affect FN during chemotherapy; however, the previous reports did not specify the reasons. Biliary stent placement itself is more likely to cause biliary tract-related inflammation, which in turn makes chemotherapy-induced FN more likely.

There were several limitations to our study. 1^st^, it was a retrospective analysis at a single institution, and the sample size was very small. In particular, since stent group had higher number of Ph cancer, some might concern the risk of bias. Therefore, we conducted the several analyses such as stratified for location of cancer (i.e., Ph cancer and Pbt cancer) or propensity matching for conducting stent insertion (i.e., location of cancer and BMI). Eventually, both stratified analysis and propensity matching analysis showed the same results that stent group had a shorter survival than non-stent group. Therefore, we interpreted that the biliary stent may reduce the survival. However, if possible, we should have considered the other variables such as resectable classification, tumor staging, and tumor size. Due to the small number of patients, increasing variables other than location of cancer and BMI further reduced the number of patients after matching, making comparisons difficult. In the results in Table 5, distant metastasis was significantly associated with longer survival (HR, 0.18; 95% CI: 0.05–0.68; *P* = .01), but we think this was clinically strange. This may be due to the small number of cases without distant metastasis even after matching. Second, we compared the effects of biliary stents on chemotherapy in patients with pancreatic cancer by evaluating the survival time; however, we could not make a direct comparison of the survival time because we included patients who were treated with mFFX as the 1^st^-line and second-line treatment in both the stent and non-stent groups, respectively, which could have resulted in survival bias. Additionally, since it was difficult to match the patients’ backgrounds, we defined chemotherapy as mFFX, used propensity score matching, and validated the definition of OS as the duration between initiation of chemotherapy and death. A biliary stent was inserted for MBO, which is unavoidable in patients with pancreatic cancer. It was not possible to follow-up whether FN and stent-related events affect the prognosis directly. The prognosis in patients who developed MBO was considered to be poor rather than considering the stent itself as detrimental to survival. Finally, we did not define the type of stent or method of stent insertion in detail.

In conclusion, in our experience, stents had a significant effect on survival in patients with advanced pancreatic cancer who received mFFX. The rate of FN was higher in the stent group than in the non-stent group. Stent insertion was an independent risk factor for overall mortality even after adjusting for other confounding factors using propensity score matching. Stent insertion is essential for relieving MBO and for safe chemotherapy, and it is important to not deprive a patient of its advantages. Therefore, we need to assess the patient’s condition with discretion and develop a treatment strategy with the short prognosis in mind after stent insertion.

## Acknowledgments

We would like to thank the patients and their families.

## Author contributions

**Conceptualization:** Akie Yoshikawa-Kimura, Koichi Taira.

**Data curation:** Akie Yoshikawa-Kimura.

**Formal analysis:** Satoko Ohfuji.

**Writing – original draft:** Akie Yoshikawa-Kimura, Koichi Taira.

**Writing – review & editing:** Akie Yoshikawa-Kimura, Koichi Taira, Hirotsugu Maruyama, Yuki Ishikawa-Kakiya, Masafumi Yamamura, Kojiro Tanoue, Atsushi Hagihara, Sawako Uchida-Kobayashi, Masaru Enomoto, Kenjiro Kimura, Shogo Tanaka, Ryosuke Amano, Shigekazu Takemura, Satoko Ohfuji, Fumio Tanaka, Yasuaki Nagami, Yasuhiro Fujiwara.

## Supplementary Material


