# Supplementary material for: Influence of a biliary stent in patients with advanced pancreatic cancer treated with modified FOLFIRINOX
Source: Medicine (Baltimore). 2022 Dec 9;101(49):e32150. doi: 10.1097/MD.0000000000032150 (PMC9750610; doi:10.1097/MD.0000000000032150)
Supplement: Supplementary file 1 [file medi-101-e32150-s001.pdf]

Supplementary content Figure 1. A flowchart from advanced pancreatic cancer patients who received modified FOLFIRINOX in our institution.

Supplemental Figure 1

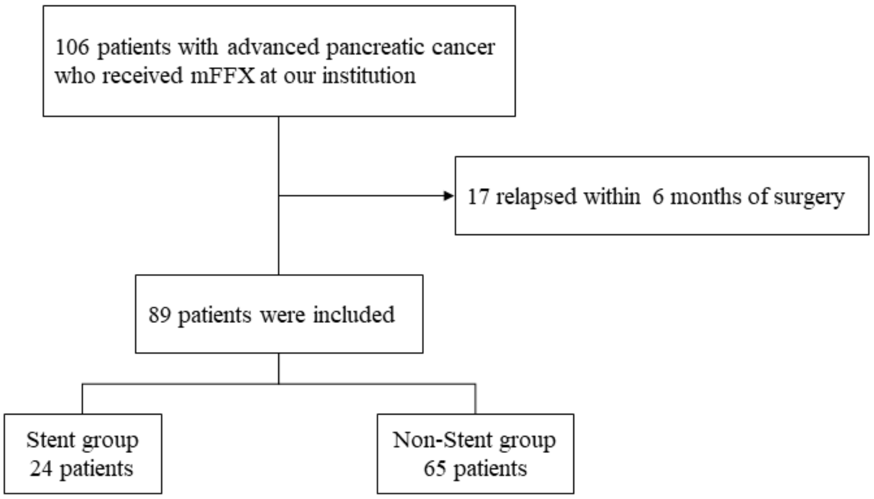

First-line treatment:	Stent group	Non-Stent group
mFFX	11	26
GnP	10	35
GS	2	3
GEM	1	1
