# Supplementary material for: Influence of a biliary stent in patients with advanced pancreatic cancer treated with modified FOLFIRINOX
Source: Medicine (Baltimore). 2022 Dec 9;101(49):e32150. doi: 10.1097/MD.0000000000032150 (PMC9750610; doi:10.1097/MD.0000000000032150)

Supplementary content Figure 2. Overall survival (OS) of stratified analysis. Stratified analysis included only patients with pancreatic head (Ph) cancer. Of the 89 patients, 20 patients each had Ph cancer in the stent and non-stent groups, respectively. The median OS was 12.1 months (95% confidence interval [CI]: 7.1–27.8) and 24.9 months (95% CI: 12.7–32.6) in the stent group and non-stent group, respectively (*P* = 0.03). On the other hand, the median OS was also shorter in the stent group than in the non-stent group in patients with pancreatic body/tail (Pbt) cancer (9.7 vs. 17.4 months; *P* = 0.02).

Supplemental Figure 2

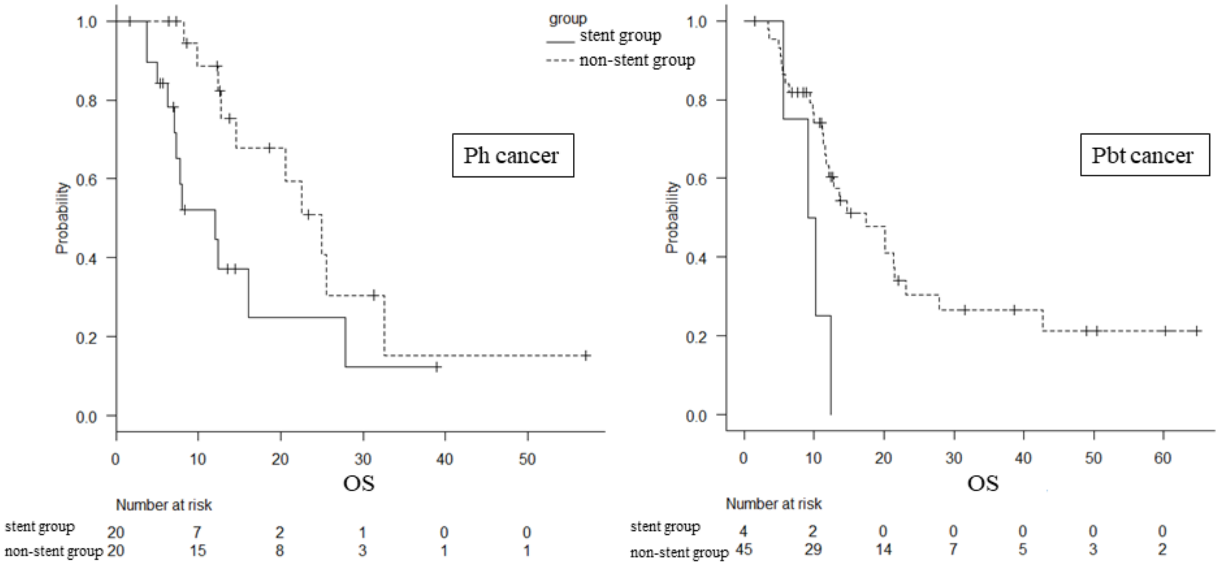

Supplement: Supplementary file 2 [file medi-101-e32150-s002.pdf]
