# Supplementary material for: Influence of a biliary stent in patients with advanced pancreatic cancer treated with modified FOLFIRINOX
Source: Medicine (Baltimore). 2022 Dec 9;101(49):e32150. doi: 10.1097/MD.0000000000032150 (PMC9750610; doi:10.1097/MD.0000000000032150)

**Supplementary Figure 3.** Overall survival (OS) in 89 patients (40 with pancreatic head [Ph] cancer and 49 with pancreatic body/tail [Pbt] cancer). The median OS was 16.1 months (95% confidence interval [CI]: 12.1–25.5) and 13.6 months (95% CI: 11.3–21.4) in Ph cancer and Pbt cancer, respectively ($P$ = 0.78).

Supplemental Figure 3

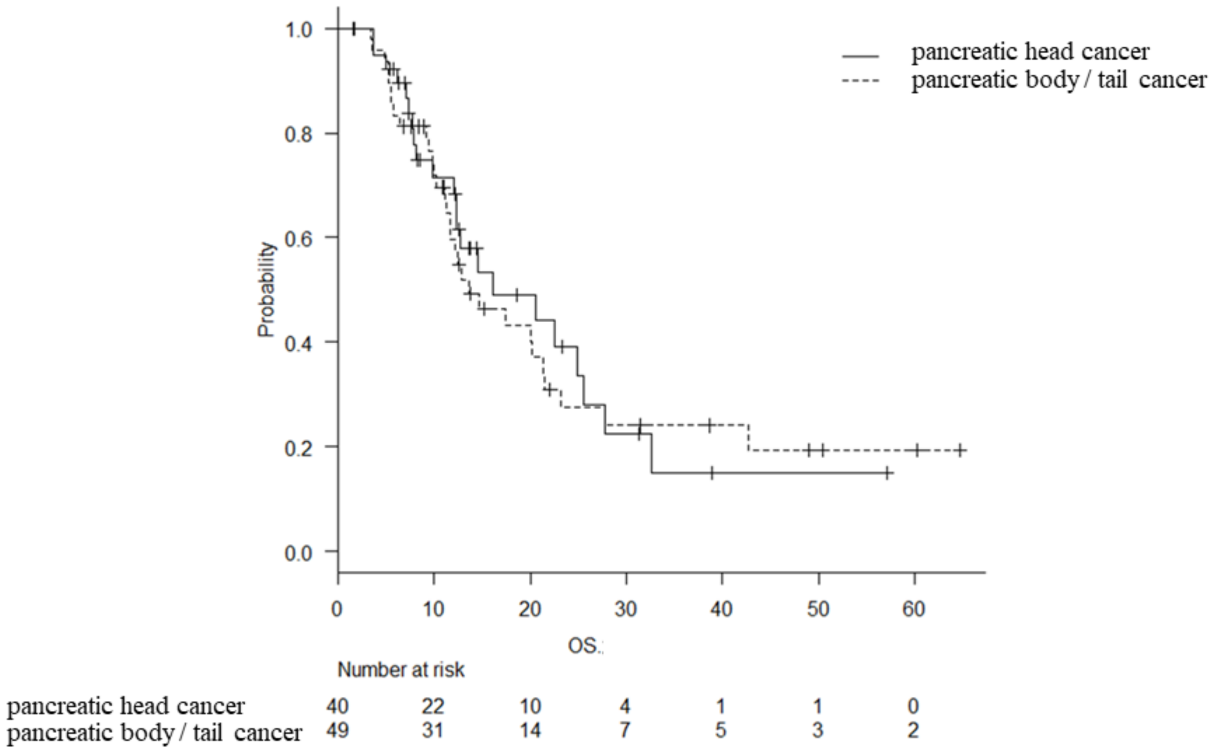

Supplement: Supplementary file 3 [file medi-101-e32150-s003.pdf]
